# Kikuchi-Fujimoto disease initially presenting with severe digestive symptoms and progressing to systemic lupus erythematosus: a case report and literature review

**DOI:** 10.3389/fped.2026.1732348

**Published:** 2026-04-02

**Authors:** Yi-De Li, Zhiwei Zhu, Hao-Dong Jiang, Qia Cheng, Man-Qiong Yang, Boyu Tan, Yu-Pin Tan

**Affiliations:** 1 Department of Radiology, Changsha Stomatological Hospital, School of Dental Medicine, Hunan University of Chinese Medicine, Changsha, Hunan, China; 2 Department of Pediatrics, Hunan Provincial People’s Hospital, The First Afffliated Hospital of Hunan Normal University, Changsha, Hunan, China; 3 Department of General Medicine, Xinhua Dong Autonomous County People’s Hospital, Huaihua, Hunan, China; 4 Department of Pharmacy, Shanghai Children’s Hospital, School of medicine, Shanghai Jiao Tong University, Shanghai, China

**Keywords:** antinuclear antibodies, Kikuchi-Fujimoto disease, lymphadenopathy, pathology, systemic lupus erythematosus

## Abstract

Kikuchi-Fujimoto disease (KFD), also known as histiocytic necrotizing lymphadenitis, is a rare idiopathic inflammatory disorder characterized by low incidence and a propensity for misdiagnosis. Although its etiology remains elusive, KFD is often linked to underlying autoimmune conditions. Definitive diagnosis requires lymph node biopsy. Classically, patients present with cervical lymphadenopathy and fever; however, this report describes an atypical, severe manifestation in a patient whose initial symptoms were dominated by prominent gastrointestinal involvement, including abdominal pain and vomiting. Histopathological evaluation of an excised lymph node confirmed the diagnosis. While corticosteroid therapy led to resolution of KFD symptoms, the patient's course was further complicated by the subsequent onset of systemic lupus erythematosus (SLE). Targeted immunosuppressive therapy for SLE ultimately achieved sustained remission. This case underscores the diagnostic pitfalls of KFD with an atypical gastrointestinal prodrome, reaffirms its established yet pivotal association with SLE, and advocates for vigilant long-term surveillance in affected individuals.

## Case presentation

**Figure 1:**
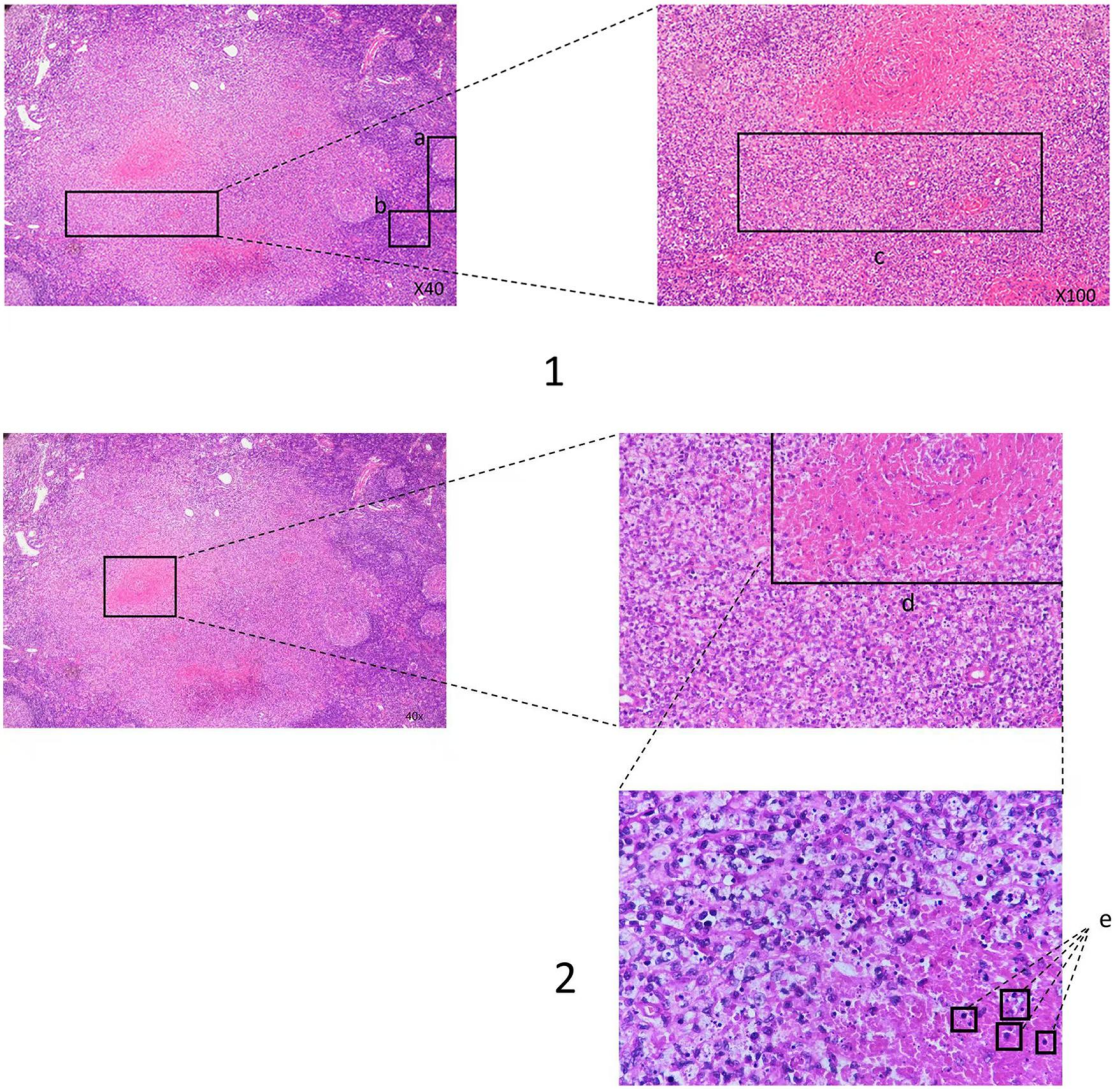
Lymph node biopsy reveals that lymph node structures are partially preserved, with follicular hyperplasia, paracortical hyperplasia, and patchy, well-defined necrotic areas. The necrotic lesions are characterized by abundant degenerative nuclear fragments and a significant accumulation of histiocytes at the borders. Crescent-shaped histiocytes are prominently observed within the necrotic areas, with small lymphocytes, activated T cells, and some plasma cells scattered among the histiocytes. Neutrophils and eosinophils are notably absent. **(1a)** represents a normal lymph node. **(1b)** indicates paracortical hyperplasia. **(1c)** shows the significant accumulation of histiocytes. **(2d)** highlights the well-defined necrotic area. **(2e)** illustrates the necrotic nuclear fragments.

A 14-year-old male patient presented with nausea, vomiting, and abdominal pain persisting for seven days and was admitted to our hospital on April 15, 2022. On physical examination, his body temperature was 36.8 °C, pulse rate was 76 beats per minute, respiratory rate was 20 breaths per minute, and blood pressure was 122/69 mmHg (1 mmHg = 0.133 kPa). No skin rashes or palpable superficial lymphadenopathy were detected, cardiopulmonary examinations showed no significant abnormalities. Mild tenderness was noted in the upper abdomen without rebound tenderness. Laboratory investigations tests were subsequently carried out. Peripheral blood count revealed a white blood cell count (WBC) of 0.65 × 10^9^/L, neutrophils (N) at 0.27 × 10^9^/L, and platelets (PLT) at 95 × 10^9^/L. Liver function tests demonstrated elevated levels of alanine transaminase (ALT) at 222.80 U/L and aspartate transaminase (AST) at 372.16 U/L. Evidence of bone marrow hyperplasia was detected. Examination of bone marrow indicated some swollen granulocytes, prominent hemophagocytosis, notable megakaryocytes, and dispersed platelets in small clusters. Conversely, bone marrow biopsy revealed hypoplasia with an approximately normal granulocyte-to-erythrocyte ratio. The granulocyte lineage was mainly comprised of myelocyte and its earlier-stage cells, with scattered myeloblasts. The erythroid lineage included mostly intermediate and late-stage erythroblasts, with occasional early-stage precursors. Megakaryocytes, mainly polymorphonuclear, were detected at a frequency of 1–3 per high-power field (HPF). No fibroblast proliferation was observed in the bone marrow stroma. Screening for hepatitis, autoimmune liver diseases, respiratory virus antigens, Epstein–Barr virus (EBV), and multiple tumor markers yielded negative results. Imaging studies, including chest radiography and gastrointestinal endoscopy, indicated no significant abnormalities. The patient was treated with omeprazole to inhibit gastric acid secretion for gastric mucosal protection, phloroglucinol for spasmolysis and pain relief, and recombinant human granulocyte colony-stimulating factor to increase peripheral neutrophil counts. Despite this, the clinical outcome was unsatisfactory. Given the multi-system involvement and unclear etiology, rheumatic connective tissue disease was suspected. Additional autoimmune tests revealed positive antinuclear antibodies (ANA, titer of 1:1,000 by indirect immunofluorescence) and a reduced serum complement C3 level of 0.74 g/L with normal C4 levels. Abdominal CT scans identified multiple small lymph nodes around the gastric fundus and the liver-gastric region, with the largest measuring 6 mm in diameter. On the eighth day of hospitalization, a soft, mobile, peanut-sized mass was palpated in the right mandible. Ultrasound confirmed this as an enlarged lymph node measuring approximately 20.5 mm × 7.6 mm. In the following day, additional enlarged lymph nodes were palpated in the left neck and left inguinal regions. Hence, a biopsy of the left cervical lymph node was performed (Figure 1). Immunohistochemistry findings revealed the following: CD30 (scattered +), CD21 (FDC network +), CD3 (+), Ki67 (+, 20%), CD123 (+), MPO (+), CD5 (−), CD68 (+), Bcl-2 (−), and Bcl-6 (−). These findings were consistent with the histopathology of histiocytic necrotizing lymphadenitis (Kikuchi-Fujimoto disease, KFD). Following the lymph node biopsy, the patient developed a fever, with the peak temperature of 39.5 °C. On the third day of fever, treatment with prednisone acetate tablets (10 mg, three times daily) was initiated. The patient showed significant improvement upon three days of treatment and was subsequently discharged. Prednisone acetate was gradually tapered off after discharge. Follow-up visits on June 23, 2022, and August 16, 2022, revealed normalized blood counts and significantly reduced transaminase levels, with the ALT at 169.4 U/L and AST at 58.1 U/L.

**Table 1:** Comprehensive laboratory test results for the pediatric patient at initial and subsequent hospital admissions.

Indicators	Reference range	First hospital admission	Second hospital admission
White blood cells (mm^3^)	4,000–11,000	650	2,010
Proportion of classification (%)			
Neutrophils	37–77	41.1	49.6
Lymphocytes	17–54	49.5	42.7
Platelets (mm^3^)	1,50,000–4,07,000	95,000	1,73,000
Hemoglobin (g/dL)	12.9–17.2	13.9	11.5
Erythrocyte Sedimentation rate (mm/h)	0–20	2	11
C-reactive protein (mg/L)	0–6	3.19	3.22
Serum creatinine (μmol/L)	37–93	44.63	64.00
Liver function			
ALT (U/L)	7–43	222.80	79.2
AST (U/L)	12–37	372.16	105.3
Albumin (g/L)	42–56	43.4	43.45
Ferritin (ng/mL)	22–275	14,370	1,320
Autoantibodies			
Antinuclear Antibody	Negative	1:1,000	1:1,000
Anti-Sm antibody	Negative	Negative	Negative
Anti-dsDNA Antibody	Negative	Negative	Negative
Complement levels			
C3 (g/L)	0.90–1.80	0.74	1.05
C4 (g/L)	0.10–0.40	0.16	0.24
Urine tests			
Urinary protein	Negative	Negative	1+
Urinary red blood cells (/HP)	0–6	0	0
Urinary white blood cells (/HP)	0–8	0	7
24 h Urinary protein (mg/24 h)	<150	<150	1,433
Blood culture	Sterile growth	Sterile growth	Sterile growth
EBV-DNA(copies/mL)	4.00E + 02	<4.00E + 02	<4.00E + 02
CMV-DNA(copies/mL)	4.00E + 02	<4.00E + 02	<4.00E + 02
T-SPOT	Negative	Negative	Negative

**Table 2:** Comparison of clinical symptoms, laboratory tests, and pathological findings between KFD and SLE.

Characters	SLE (%)	KFD (%)
Clinical symptoms		
Cervical Lymphadenopathy	40%	50%–98%
Generalized Lymphadenopathy	10%	1%–22%
Fever	52%	30%–67%
Weight loss	83%	10%–51%
Hepatosplenomegaly	10%–45%	1%–22%
Arthralgia/arthritis	60%/20%	5%–34%
Headache	25%–80%	17%–33%
Diarrhea	Rare	Rare
Skin lesions	53%–78%	10%–40%
Laboratory tests		
Anemia	60%–70%	28%–54%
Leukopenia/lymphopenia	30%–40%	25%–58%
Thrombocytopenia	25%–50%	5%
ANA positive	70%–98%	8%–45%
Anti-dsDNA positive	70%	7%–18%
Decreased complement levels	47%–55%	21%–27%
Pathological examination		
Histopathological examination	Follicular hyperplasia, fragmented immunoblasts, plasma cells, increased vasculature, and Azzopardi phenomenon	Subcapsular expansion with distinct, patchy necrotic areas containing amorphous eosinophilic and apoptotic fragments. Necrotic areas surrounded by abundant crescentic histiocytes and varying numbers of immunoblasts. Clustered plasma cell-like dendritic cells may also be observed.
Immunohistochemistry	CD4+ T and CD8+ T lymphocytes	CD8+ T lymphocytes, myeloperoxidase+, CD68+, CD123+

**Figure 2:**
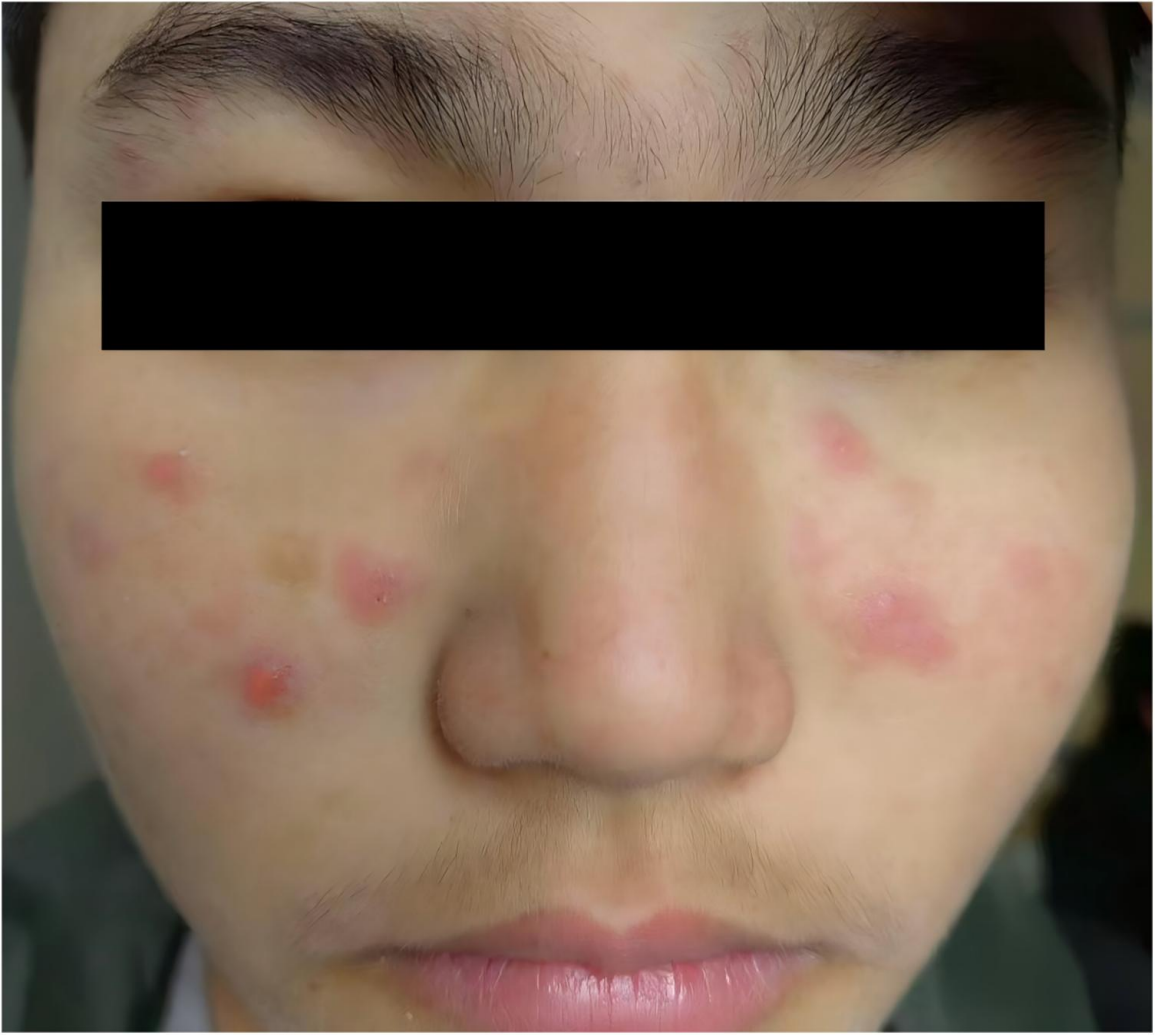
Erythema on both cheeks and the face of the patient.

**Figure 3:**
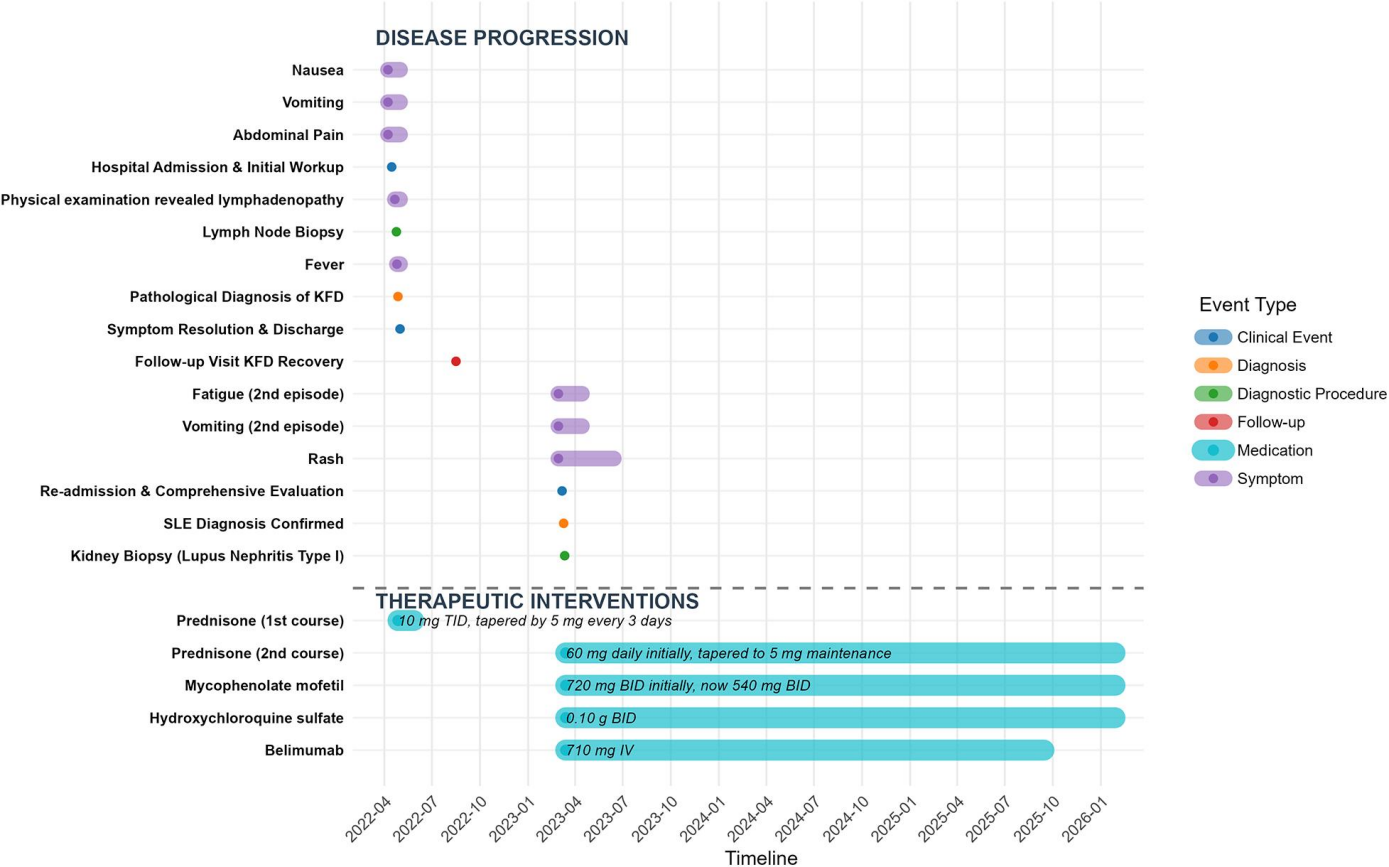
Timeline of key clinical events and therapeutic interventions for the patient.

On March 7, 2023, the patient was readmitted for complaint of one-week history of fatigue and vomiting. Physical examination revealed erythema on both cheeks and the face (Figure 2), along with multiple palpable, soft, mobile lymph nodes in the neck and lower jaw, with the largest measuring approximately 20 mm in diameter. Laboratory tests revealed a WBC of 2.01 × 10^9^/L and Neutrophils at 0.99 × 10^9^/L, while red blood cells and PLT counts within normal ranges. Liver function tests indicated ALT of 79.2 U/L and an AST of 105.3 U/L. Although KFD recurrence was initially suspected, the presence of facial rashes raised concern for SLE, prompting further autoimmune testing. The results revealed positive ANA (titer 1:1,000), positive anti-Sm antibodies as well as positive anti-nRNP/Sm antibodies. According to the 2019 classification criteria for SLE (2019 EULAR/ACR), the patient scored a total of 13 points: 3 points for leukopenia, 6 points for positive anti-Sm antibodies, and 4 points for subacute cutaneous lupus rash, confirming the diagnosis of SLE. Additionally, the patient's 24 h urine protein was 1,433 mg. A subsequent kidney biopsy revealed mild lupus nephritis (Type I). The detailed laboratory test results of the patient during the two admissions are shown in (Table 1). Standard SLE therapy (prednisone acetate 60 mg daily, mycophenolate mofetil 720 mg twice daily, hydroxychloroquine sulfate 0.10 g twice daily, and belimumab 710 mg intravenously) was then administered. Following treatment, the patient showed significant improvement and was discharged. Currently followed up for almost 3 years, the patient was stable without any clinical symptoms. SLEDAI-2000 Score is 0, with negative autoantibodies, normal complement and urinary protein levels. Belimumab was discontinued and current treatment includes prednisone acetate (5 mg daily), mycophenolate mofetil (540 mg twice daily) and hydroxychloroquine sulfate (0.10 g twice daily).Therefore, a timeline summarizing the patient's clinical course and interventions is presented (Figure 3).

## Discussion

The exact cause and mechanism of KFD remains unclear, although viral infections or autoimmune responses are suspected to play a role (1–3). KFD is a self-limiting condition characterized by leukopenia and a lack of response to antibiotic treatment, which supports a potential viral etiology. A study indicated that viruses such as EBV and herpes simplex virus may be implicated (4–6). Most patients with KFD exhibit negative serological markers for autoimmune diseases, such as ANA and anti-double-stranded DNA antibodies, with only a minority showing positive results (7–9). In this case, the patient initially presented with positive ANA (titer of 1:1,000) but did not meet the diagnostic criteria for SLE. A diagnosis of KFD was established through lymph node biopsy. Notably, the progression of KFD to autoimmune conditions, particularly SLE, has been reported in previous literature (8, 10, 11). The patient was subsequently diagnosed with SLE during a second hospitalization. Nevertheless, the precise mechanism underlying the progression from KFD to autoimmune diseases remains poorly understood and warrants further investigation. It is also possible that the patient initially presented with both KFD and SLE, though the evidence of SLE may not have been apparent at the time. The diagnostic process for SLE can be lengthy, often taking 1–2 years or more to confirm (12–15). This highlights the importance of close monitoring in patients with KFD who test positive for autoimmune markers such as ANA or anti-double-stranded DNA antibodies. In this case, the presence of a high-titer ANA and low complement C3 at the initial KFD presentation may not have been a mere coincidence but rather an early immunological signature of a concomitant, subclinical autoimmune process. This could suggest that in some patients, KFD and SLE may represent different phases on a spectrum of an aberrant immune response, possibly triggered by an unknown viral or environmental stimulus in a genetically predisposed individual. The KFD episode could have been an intense, organ-specific (lymph node) inflammatory manifestation that preceded the full systemic evolution into classifiable SLE. Early detection and intervention are crucial, as these patients may have an increased risk of developing autoimmune diseases or coexisting inflammatory conditions.

KFD lacks specific clinical features and typically follows an acute or subacute course lasting for several weeks. Most patients present with posterior cervical lymphadenopathy (60%–90%), often with involvement of axillary and/or supraclavicular lymph nodes (16–19). The affected lymph nodes are typically firm and tender. Lymphadenopathy is commonly associated with fever with other nonspecific symptoms including weight loss, nausea, vomiting, fatigue, headache, and joint pain (4, 20–23). Skin lesions commonly associated with KFD frequently involve the face and upper body, presenting as rashes, nodules, erythematous papules, erythema induratum, or erythema multiforme (24–26). In this case, cervical lymphadenopathy was not identified in the first place. However, CT imaging revealed multiple small lymph nodes around the gastric fundus and liver, with enlarged lymph nodes palpated only in the right mandible by the 8th day of hospitalization. This pattern suggests that lymphadenopathy may have originated from internal organs and progressively involved superficial lymph nodes. The patient's gastrointestinal symptoms could be attributed to irritation of the gastrointestinal tract by the enlarged perigastric lymph nodes. Notably, some cases of gastrointestinal lymphadenopathy may present without noticeable clinical symptoms, potentially delaying medical consultation and diagnosis. KFD is a rare condition, and diagnosis relies on lymph node biopsy (27). Consequently, some patients may remain undiagnosed if they resolve spontaneously before the appearance of superficial lymphadenopathy. Laboratory test results in KFD are generally unremarkable, though mild anemia, slightly elevated C-reactive protein, and increased erythrocyte sedimentation rate have been reported in some cases (3, 18, 28). Other laboratory findings may include leukopenia, increased lactate dehydrogenase, and elevated transaminases (12, 20, 28, 29). Additionally, atypical lymphocytes can be observed in the peripheral blood of up to one-third of patients.

The diagnosis of KFD is primarily based on lymph node pathology and morphology. The clinical manifestations of KFD often closely resemble those of SLE, making differentiation between the two challenging based solely on lymph node morphology. Histologically, KFD typically shows partially preserved lymph node structures with follicular hyperplasia and an expanded paracortical area containing scattered, well-defined necrotic zones. The necrotic areas are characterized by abundant fragmented nuclear debris, with a notable accumulation of histiocytes at the borders. Additionally, the necrotic lesions in KFD also contain numerous crescent-shaped histiocytes, interspersed with scattered small lymphocytes, activated T cells, and some plasma cells (30). Notably, neutrophils and eosinophils are generally absent. The borders of the necrotic areas may show clusters of plasma cell-like dendritic cells and immunoblasts, along with blood vessels forming thrombi. In contrast, the most distinctive histological feature of SLE-related lymphadenopathy is the presence of hematoxylin bodies, although these are observed in only a small proportion of SLE patients (7). In this case, the patient did not meet the diagnostic criteria for SLE during the initial visit and was diagnosed with KFD based on lymph node biopsy findings. During the second hospitalization, the patient met the diagnostic criteria for SLE; however, no further lymph node biopsy was performed, precluding a direct comparison of lymph node pathology between KFD and SLE. Further research and case studies are warranted to better understand the histopathological differences and similarities between KFD and SLE-related lymphadenopathy. Notably, KFD has been associated with autoimmune diseases (31–35). A previous study reported that approximately 30% of KFD cases occurred prior to an SLE diagnosis, while around 23% of cases were diagnosed with SLE after KFD was recognized (36). The differential diagnostic features of KFD and SLE are summarized in (Table 2).

In conclusion, KFD is a rare, benign, self-limiting condition, although it has the potential to progress to other autoimmune diseases, particularly SLE. In patients with KFD who test positive for autoimmune markers, close follow-up is essential to monitor the development of autoimmune disease or the coexistence of self-limiting inflammatory conditions.

## Data Availability

The original contributions presented in the study are included in the article/Supplementary Material, further inquiries can be directed to the corresponding author/s.
